# The combined associations of depression and cognitive impairment with functional disability and mortality in older adults: a population-based study from the NHANES 2011–2014

**DOI:** 10.3389/fnagi.2023.1121190

**Published:** 2023-05-04

**Authors:** Shuang Han, Yue Gao, Da Gan

**Affiliations:** ^1^Zhejiang Key Laboratory of Traditional Chinese Medicine for the Prevention and Treatment of Senile Chronic Diseases, Department of Geriatrics, Affiliated Hangzhou First People’s Hospital, Zhejiang University School of Medicine, Hangzhou, Zhejiang, China; ^2^Chronic Disease Research Institute, The Children’s Hospital, and National Clinical Research Center for Child Health, School of Public Health, School of Medicine, Zhejiang University, Hangzhou, Zhejiang, China; ^3^Department of Nutrition and Food Hygiene, School of Public Health, School of Medicine, Zhejiang University, Hangzhou, Zhejiang, China

**Keywords:** depression, cognitive impairment, functional disability, mortality, older adults

## Abstract

**Objective:**

The present study aimed to explore the combined associations of depression and cognitive impairment with functional disability and mortality, and whether the joint effects of depression and cognitive impairment on mortality were influenced by functional disability.

**Methods:**

A total of 2,345 participants aged 60 and above from the 2011–2014 cycle of the National Health and Nutrition Examination Survey (NHANES) were included in the analyses. Questionnaires were used to evaluated depression, global cognitive function and functional disability (including disability in activities of daily living (ADLs), instrumental activities of daily living (IADLs), leisure and social activities (LSA), lower extremity mobility (LEM), and general physical activity (GPA)). Mortality status was ascertained up to December 31, 2019. Multivariable logistic regression was performed to investigate the associations of depression and low global cognition with functional disability. Cox proportional hazards regression models were conducted to evaluate the effect of depression and low global cognition on mortality.

**Results:**

Interactions between depression and low global cognition were observed when exploring associations of depression and low global cognition with IADLs disability, LEM disability, and cardiovascular mortality. Compared with normal participants, participants with both depression and low global cognition had the highest odds ratios of disability in ADLs, IADLs, LSA, LEM, and GPA. Besides, participants with both depression and low global cognition also had the highest hazard ratios of all-cause mortality and cardiovascular mortality, and these associations remained after adjusting for disability in ADLs, IADLs, LSA, LEM, and GPA.

**Conclusion:**

Older adults with both depression and low global cognition were more likely to have functional disability, and had the highest risk of all-cause mortality and cardiovascular mortality.

## Introduction

Depression and cognitive impairment are two common disorders among the older adults (Polyakova et al., 2014). The prevalence of depression and cognitive impairment were reported to be 11.2 and 18.6% among the US older adults, respectively (Steffens et al., 2009; Hale et al., 2020). Emerging evidence indicates that depression and cognitive impairment are closely intertwined, and these two symptoms can be mutually prompted (Scult et al., 2017; Zheng et al., 2018).

Functional ability–the ability to conduct physical activities of daily living—is helpful for older individuals to live independently (Kuo et al., 2007). Studies found that functional disability was associated with an increased risk of poor quality of daily life and premature death (WHO, 2001; Murad et al., 2015; Wu et al., 2016), which highlighted the importance of identifying risk factors for functional disability.

Previous studies reported that depression and cognitive impairment were associated with functional disability (Mehta et al., 2002; Yochim et al., 2008; Li and Conwell, 2009; Olaya et al., 2016; Wu, 2021; Yang et al., 2021). However, only a few studies have explored the combined associations of depression and cognitive impairment with functional disability, and the results were inconsistent (Mehta et al., 2002; Li and Conwell, 2009; Olaya et al., 2016; Wu, 2021). A longitudinal study conducted in older adults found that improvement of depression could further attenuate the adverse effect of cognitive impairment on instrumental activities of daily living disability (Li and Conwell, 2009). In contrast, the other two prospective studies found that the interactive effect between depression and cognitive impairment not existed for disability in activities of daily living or disability in instrumental activities of daily living disability (Mehta et al., 2002; Wu, 2021). In addition, a community-based study of 7,987 older adults found that country-specific difference is implicated in the combined associations of depression and cognitive impairment with functional disability (Olaya et al., 2016). They found that the interactive effect between depression and cognitive impairment on disability only existed in older adults from Finland but not in older adults from Poland and Spain, and the direction of interaction effects contradicted the previous study (Olaya et al., 2016).

Furthermore, few studies have investigated the joint effects of depression and cognitive impairment on mortality, and it is not well understood whether these effects were affected by functional disability (Arfken et al., 1999; Georgakis et al., 2016).

Hence, the present study aimed to investigate the combined associations of depression and cognitive impairment with functional disability and mortality, and whether the joint effects of depression and cognitive impairment on mortality were influenced by functional disability.

## Methods

### Study participants

The National Health and Nutrition Examination Survey (NHANES) used a stratified, multistage, probability cluster sampling design to assess the nationally representative health and nutritional status of the non-institutionalized US civilian. The present study included data from 3,632 adults aged 60 and above from the NHANES (2011–2012 and 2013–2014). The present study further excluded 1,287 participants with missing information on depression, cognitive function assessment, and functional disability. Finally, a total of 2,345 participants were included in the analyses.

The NHANES procedures and protocols were approved by the National Center for Health Statistics Research Ethics Review Board. All participants provided written informed consent for participation in the NHANES (Parsons et al., 2014). More information on the NHANES is available on: https://www.cdc.gov/nchs/nhanes/index.htm.

### Definitions of depression

Depression was assessed using the Nine-item Patient Health Questionnaire (PHQ-9), which was widely used for screening for non-psychiatric depression (Kroenke et al., 2001). PHQ-9 has 9 items and each item scored from 0 (“not at all”) to 3 (“nearly every day”). The total PHQ-9 score ranges from 0 to 27, and participants with a total PHQ-9 score ≥ 10 were diagnosed as having major depression. This cutoff has a sensitivity of 88% and a specificity of 88% for diagnosing major depression (Kroenke et al., 2001).

### Definitions of low global cognition

Cognitive function was evaluated using the Consortium to Establish a Registry for Alzheimer’s Disease (CERAD), the Animal Fluency test, and the Digit Symbol Substitution Test (DSST; Morris et al., 1989; Wechsler, 1997; Strauss et al., 2006). The CERAD test consists of three consecutive learning trials and one delayed recall, which was used to assess the immediate and delayed learning ability for new verbal information. The score of each trial ranges from 0 to 10, and the total scores range from 0 to 40 (Morris et al., 1989). The Animal Fluency test was conducted to examine categorical verbal fluency, with the total scores ranging from 0 to 40 (Strauss et al., 2006). The Digit Symbol Substitution test (DSST) was performed to evaluate processing speed, visual scanning, sustained attention, and short-term memory, with the scores ranging from 0 to 133 (Wechsler, 1997). Higher scores of the three tests indicated better cognitive performance.

Then, the scores of each cognitive test were Z-score transformed. The overall cognitive score was calculated as the average of the standardized scores from three cognitive tests. Because there is no well-defined threshold for identifying low cognitive performance, we defined participants with an overall cognitive score in the lowest unweighted quartile in the study population as having low global cognition (Brody et al., 2019; Huang and Ren, 2022).

### Definitions of functional disability

Functional disability was evaluated using the 19-item physical functioning questionnaire (Cosiano et al., 2020). Physical functioning consisted of five main domains: (Polyakova et al., 2014) activities of daily living (ADLs) (getting in and out of bed, eating, and dressing); (Steffens et al., 2009) instrumental activities of daily living (IADLs) (managing money, performing house chores, preparing meals); (Hale et al., 2020) leisure and social activities (LSA) (going to the movies, attending social events, performing leisure activity at home); (Scult et al., 2017) lower extremity mobility (LEM) (walking a quarter mile, walking up to 10 steps, walking between rooms on the same floor); and (Zheng et al., 2018) general physical activity (GPA) (lifting or carrying heavy objects, reaching up overhead, grasping/holding small objects, standing for long periods, sitting for long periods). The answer to each item was “no difficulty,” “some difficulty,” “much difficulty” or “unable to do.” Participants were defined as having a functional disability in this domain if some difficulty in one or more items was reported in a given domain (Cosiano et al., 2020).

### Mortality

Mortality status and cause of death were identified by linking to death records in the National Death Index public access files through December 31, 2019 (Statistics NCfH, 2021). Cardiovascular mortality and cancer mortality were ascertained based on the underlying causes of death from the International Classification of Diseases codes.

### Covariate

Information on age (60–64, 65–69, 70–74, 75–79, and 80+), gender (male and female), race (Mexican American, other Hispanic, non-Hispanic White, non-Hispanic Black, and other race), marital status (never married, married or living with a partner, and widowed or separated or divorced), education (less than high school, high school, and college education or above), smoke (never, former, and current), alcohol drinking (yes and no), and social economic status were obtained by questionnaire survey. A modified Global Physical Activity Questionnaire was used to measure physical activity and participants were classified as physically active if their consumed metabolic equivalent scores (MET) ≥ 600 MET-minutes/week. Social economic status was classified using the ratio of family income to poverty (PIR) as low (PIR ≤ 1.3), middle (PIR > 1.3 to ≤1.85) or high (PIR > 1.85). Body mass index (BMI) was categorized as underweight (< 18.5 kg/m^2^), normal (18.5–24.9 kg/m^2^), overweight (25.0–29.9 kg/m^2^), obese class I (30–34.9 kg/m^2^), and obese class II or class III (≥ 35 kg/m^2^).

Participants with a self-reported history of coronary heart disease (CHD), angina, or myocardial infarction were defined as having CHD. Diabetes was ascertained if participants had a self-reported diagnosis of diabetes, were taking medicine for diabetes, or had glycosylated hemoglobin ≥6.5%. Hypertension was determined if participants reported a history of hypertension, were taking prescription for hypertension, or had a mean systolic blood pressure ≥ 140 mm Hg or a mean diastolic blood pressure ≥ 90 mm Hg. Stroke was defined if participants had a history of stroke. Cancer was defined if participants had a history of cancer or malignancy. Comprehensive descriptions of methodology and data collection were provided elsewhere (Prevention CfDCa, n.d.).

### Statistical analyzes

According to the status of depression and global cognition, participants were classified into four groups: normal, depression only, low global cognition only, and coexistence of depression and low global cognition. Differences in baseline characteristics between groups were compared using weighted chi-square tests.

We conducted weighted multivariate logistic regression models to explore the separate and combined associations of depression and low global cognition with each component of functional disability, and interactions between depression and low global cognition were examined. Age categories, gender, race, marital status, education, smoke, alcohol drinking, physical activity, PIR category, BMI category, CHD, diabetes, hypertension, stroke, and cancer were adjusted in the regression models.

Kaplan–Meier (weighted) analysis was conducted to describe the survival of participants with different status of depression and global cognition. Weighted Cox proportional hazards regression models were performed to evaluate the separate and joint effect of depression and low global cognition on all-cause mortality, cardiovascular mortality, and cancer mortality. Age categories, gender, race, marital status, education, smoke, alcohol drinking, physical activity, PIR category, BMI category, CHD, diabetes, hypertension, stroke, and cancer were adjusted in the Cox model 1. To further explore whether the joint effects of depression and low global cognition on all-cause mortality, cardiovascular mortality and cancer mortality were influenced by functional disability, we additionally adjusted for disability in ADLs, IADLs, LSA, LEM, and GPA based on Cox model 1 (Cox model 2).

All statistical analyzes were conducted using STATA 15.0. A two-sided *p*-value of <0.05 was considered statistically significant.

## Results

The characteristics of participants with different status of depression and low global cognition are shown in Table 1. Among 2,345 participants, 112 (4.8%) participants had depression only, 499 (21.3%) participants had low global cognition only, and 88 (3.8%) participants had both depression and low global cognition. Significant differences in age categories, gender, race, marital status, education, alcohol drinking, physical activity, PIR category, prevalence of CHD, diabetes, hypertension, and stroke, and prevalence of disability in ADLs, IADLs, LSA, LEM, and GPA were observed between groups.

**Table 1:** Characteristics of participants with different status of depression and low global cognition.

Variable	Normal	Depression only	Low global cognition only	Coexistence of depression and low global cognition	*P*
Number of participants	1,646	112	499	88	
Age categories, years					<0.001
60–64	599 (36.7%)	53 (51.8%)	95 (13.8%)	26 (23.2%)	
65–69	407 (26.6%)	28 (22.2%)	100 (13.1%)	29 (28.7%)	
70–74	312 (18.7%)	17 (16.6%)	105 (23.1%)	12 (13.5%)	
75–79	154 (9.2%)	6 (3.4%)	67 (17.3%)	4 (4.2%)	
80+	174 (8.9%)	8 (6.1%)	132 (32.7%)	17 (30.4%)	
Gender (Male, %)	744 (43.8%)	38 (33.3%)	295 (52.7%)	30 (33.5%)	0.007
Race					<0.001
Mexican American	119 (2.4%)	14 (4.6%)	48 (5.6%)	14 (9.8%)	
Other Hispanic	130 (2.4%)	15 (4.6%)	72 (8.2%)	22 (15.6%)	
Non-Hispanics White	886 (84.7%)	53 (77.7%)	176 (64.1%)	26 (54.0%)	
Non-Hispanics Black	337 (6.1%)	23 (8.3%)	157 (16.0%)	23 (17.0%)	
Other Race	174 (4.6%)	7 (4.7%)	46 (6.2%)	3 (3.5%)	
Marital Status					<0.001
Never married	88 (4.1%)	11 (7.2%)	26 (3.7%)	6 (5.5%)	
Married, living with partner	1,016 (68.9%)	52 (52.1%)	285 (59.0%)	27 (32.6%)	
Widowed, separated, or divorced	539 (27.0%)	49 (40.8%)	188 (37.3%)	55 (61.9%)	
Unknown	3 (0.1%)	0	0	0	
Education					<0.001
Less than high school	223 (9.7%)	32 (19.7%)	241 (37.8%)	57 (56.3%)	
High school	382 (20.7%)	25 (20.0%)	115 (26.5%)	19 (27.0%)	
College education or above	1,040 (69.6%)	55 (60.3%)	142 (35.6%)	12 (16.7%)	
Unknown	1 (0%)	0	1 (0.1%)	0	
Smoke					0.757
Never	850 (50.7%)	51 (43.2%)	244 (50.3%)	44 (52.0%)	
Current	184 (9.9%)	21 (16.4%)	64 (12.2%)	16 (13.8%)	
Former	611 (39.4%)	40 (40.4%)	191 (37.5%)	28 (34.2%)	
Unknown	1 (0%)	0	0	0	
Alcohol drinking					<0.001
No	484 (24.5%)	30 (26.7%)	187 (37.3%)	40 (44.3%)	
Yes	1,162 (75.5%)	81 (73.0%)	311 (62.5%)	48 (55.7%)	
Unknown	0	1 (0.3%)	1 (0.1%)	0	
Physical activity					<0.001
Active	918 (57.8%)	39 (40.4%)	210 (40.9%)	21 (18.8%)	
Inactive	725 (42.0%)	71 (58.9%)	289 (59.1%)	67 (81.2%)	
Unknown	3 (0.2%)	2 (0.6%)	0	0	
Body mass index (kg/m^2^)					0.071
< 18.5	20 (1.3%)	3 (1.6%)	14 (3.4%)	0	
18.5–24.9	437 (25.5%)	20 (20.4%)	129 (25.9%)	21 (21.1%)	
25.0–29.9	591 (36.2%)	27 (28.3%)	180 (38.0%)	25 (32.2%)	
30–34.9	362 (22.4%)	36 (28.6%)	96 (17.3%)	20 (22.0%)	
≥ 35	220 (13.5%)	26 (21.1%)	64 (12.4%)	19 (18.7%)	
Unknown	16 (1.1%)	0	16 (2.9%)	3 (6.0%)	
Family monthly poverty level index category					<0.001
≤ 1.3	346 (13.2%)	51 (29.4%)	195 (30.0%)	45 (45.9%)	
1.3–1.85	187 (9.0%)	14 (19.8%)	80 (16.3%)	15 (21.8%)	
> 1.85	1,024 (73.4%)	43 (48.9%)	191 (48.4%)	18 (21.8%)	
Unknown	89 (4.3%)	4 (1.9%)	33 (5.3%)	10 (10.6%)	
Coronary heart disease (yes, %)					0.019
No	1,462 (88.2%)	87 (77.8%)	420 (82.4%)	63 (73.3%)	
Yes	180 (11.7%)	23 (21.5%)	78 (17.4%)	24 (26.0%)	
Unknown	4 (0.1%)	2 (0.7%)	1 (0.3%)	1 (0.7%)	
Diabetes (yes, %)					<0.001
No	1,194 (76.9%)	64 (71.4%)	300 (65.3%)	47 (56.3%)	
Yes	375 (18.6%)	43 (26.6%)	169 (30.3%)	37 (39.3%)	
Unknown	77 (4.4%)	5 (2.0%)	30 (4.4%)	4 (4.4%)	
Hypertension (yes, %)					0.001
No	541 (36.5%)	27 (30.0%)	106 (20.4%)	20 (21.1%)	
Yes	1,087 (62.5%)	84 (68.0%)	391 (79.4%)	67 (77.1%)	
Unknown	18 (1.0%)	1 (2.0%)	2 (0.2%)	1 (1.8%)	
Stroke (yes, %)					<0.001
No	1,576 (95.7%)	101 (90.8%)	455 (90.4%)	75 (82.4%)	
Yes	68 (4.2%)	10 (8.6%)	44 (9.6%)	13 (17.6%)	
Unknown	2 (0.1%)	1 (0.5%)	0	0	
Cancer (yes, %)					0.653
No	1,300 (75.6%)	92 (76.8%)	414 (78.1%)	71 (81.5%)	
Yes	345 (24.4%)	20 (23.2%)	85 (21.9%)	16 (18.1%)	
Unknown	1 (0%)	0 (0%)	0	1 (0.4%)	
Component of functional disability (yes, %)					
Activities of daily living	183 (8.7%)	41 (27.1%)	101 (20.5%)	51 (57.5%)	<0.001
Instrumental activities of daily living	236 (12.6%)	51 (28.6%)	123 (26.1%)	61 (71.6%)	<0.001
Leisure and social activities	164 (8.3%)	49 (30.2%)	97 (19.7%)	51 (61.7%)	<0.001
Lower extremity mobility	383 (20.9%)	63 (44.7%)	164 (37.3%)	66 (80.2%)	<0.001
General physical activities	627 (36.3%)	83 (64.1%)	235 (50.1%)	74 (84.8%)	<0.001
Mortality (yes, %)					
All-cause mortality	195 (11.0%)	17 (9.2%)	147 (34.9%)	29 (43.2%)	<0.001
Cardiovascular mortality	60 (3.0%)	6 (2.2%)	50 (12.2%)	14 (19.5%)	<0.001
Cancer mortality	58 (3.9%)	4 (2.0%)	35 (7.8%)	4 (3.3%)	0.019

Frequency (weighted percentages) was presented for categorical variables.

The separate associations of depression and low global cognition with each component of functional disability are shown in Table 2. Both depression and low global cognition were positively associated with disability in ADLs, IADLs, LSA, and LEM, and depression was positively associated with disability in GPA. Significant interactions between depression and low global cognition were observed for disability in IADLs (*P* for interaction = 0.008) and LEM (*P* for interaction = 0.019).

**Table 2:** Separate associations of depression and low global cognition with each component of functional disability.

Functional disability	Depression		Low global cognition		The interaction effect	
	OR (95% CI)	*P*	OR (95% CI)	*P*	OR (95% CI)	*P*
ADLs	3.76 (2.27, 6.23)	<0.001	2.05 (1.39, 3.00)	0.001	1.45 (0.75, 2.83)	0.260
IADLs	2.96 (1.91, 4.58)	<0.001	2.00 (1.39, 2.89)	0.001	3.02 (1.37, 6.68)	0.008
LSA	4.10 (3.15, 5.34)	<0.001	2.07 (1.40, 3.06)	0.001	1.49 (0.67, 3.29)	0.318
LEM	2.96 (1.68, 5.20)	<0.001	1.53 (1.05, 2.23)	0.028	3.00 (1.22, 7.42)	0.019
GPA	2.88 (1.78, 4.64)	<0.001	1.20 (0.95, 1.54)	0.128	1.71 (0.55, 5.35)	0.344

Adjusted for age categories, gender, race, marital status, education, smoke, alcohol drinking, physical activity, ratio of family income to poverty category, body mass index category, coronary heart disease, diabetes, hypertension, stroke and cancer.

ADLs, activities of daily living; IADLs, instrumental activities of daily living; LSA, leisure and social activities; LEM, lower extremity mobility; GPA, general physical activity; OR, odds ratio; CI, confidence interval.

The combined associations of depression and low global cognition with each component of functional disability are shown in Table 3. Compared with normal group, depression only, low global cognition only and coexistence of depression and low global cognition were positively associated with disability in ADLs, IADLs, and LSA, and the odd ratios (ORs) were highest in participants with both depression and low global cognition. Compared with normal group, depression only and coexistence of depression and low global cognition were positively associated with disability in LEM and GPA, and the ORs were highest in the coexistence of depression and low global cognition group.

**Table 3:** Combined associations of depression and low global cognition with each component of functional disability.

Functional disability	Normal		Depression only		Low global cognition only		Coexistence of depression and low global cognition	
	OR (95% CI)	*P*	OR (95% CI)	*P*	OR (95% CI)	*P*	OR (95% CI)	*P*
ADLs	1.00 (reference)	/	3.31 (1.91, 5.75)	<0.001	1.92 (1.30, 2.85)	0.002	9.27 (4.87, 17.63)	<0.001
IADLs	1.00 (reference)	/	2.10 (1.22, 3.62)	0.009	1.73 (1.17, 2.56)	0.007	11.01 (5.68, 21.32)	<0.001
LSA	1.00 (reference)	/	3.61 (2.49, 5.24)	<0.001	1.94 (1.34, 2.82)	0.001	10.41 (5.42, 19.98)	<0.001
LEM	1.00 (reference)	/	2.20 (1.14, 4.23)	0.020	1.37 (0.92, 2.06)	0.121	9.06 (4.58, 17.92)	<0.001
GPA	1.00 (reference)	/	2.54 (1.40, 4.60)	0.003	1.16 (0.89, 1.50)	0.266	5.02 (2.04, 12.32)	0.001

Adjusted for age categories, gender, race, marital status, education, smoke, alcohol drinking, physical activity, ratio of family income to poverty category, body mass index category, coronary heart disease, diabetes, hypertension, stroke and cancer.

ADLs, activities of daily living; IADLs, instrumental activities of daily living; LSA, leisure and social activities; LEM, lower extremity mobility; GPA, general physical activity; OR, odds ratio; CI, confidence interval.

Information on death was successfully followed up for 2,341 participants, and 388 participants died during the follow-up. The median follow-up time for all participants was 6.5 years. The separate effects of depression and low global cognition on all-cause mortality, cardiovascular mortality, and cancer mortality are shown in Table 4. Low global cognition but not depression was associated with increased hazard ratios (HRs) for all-cause mortality and cardiovascular mortality. Neither depression nor low global cognition was associated with cancer mortality. A significant interaction between depression and low global cognition was observed for cardiovascular mortality (*P* for interaction = 0.037).

**Table 4:** Hazard ratios of depression and low global cognition for all-cause mortality, cardiovascular mortality and cancer mortality.

Mortality	Depression		Low global cognition		The interaction effect	
	HR (95% CI)	*P*	HR (95% CI)	*P*	HR (95% CI)	*P*
All-cause mortality	1.39 (0.80, 2.41)	0.237	1.76 (1.31, 2.35)	<0.001	2.18 (0.73, 6.49)	0.156
Cardiovascular mortality	1.89 (0.94, 3.79)	0.073	1.95 (1.17, 3.24)	0.012	4.35 (1.09, 17.25)	0.037
Cancer mortality	0.74 (0.28, 1.96)	0.536	1.27 (0.64, 2.51)	0.476	1.39 (0.26, 7.27)	0.690

Adjusted for age categories, gender, race, marital status, education, smoke, alcohol drinking, physical activity, ratio of family income to poverty category, body mass index category, coronary heart disease, diabetes, hypertension, stroke and cancer.

HR, hazard ratio; CI, confidence interval.

**Figure 1:**
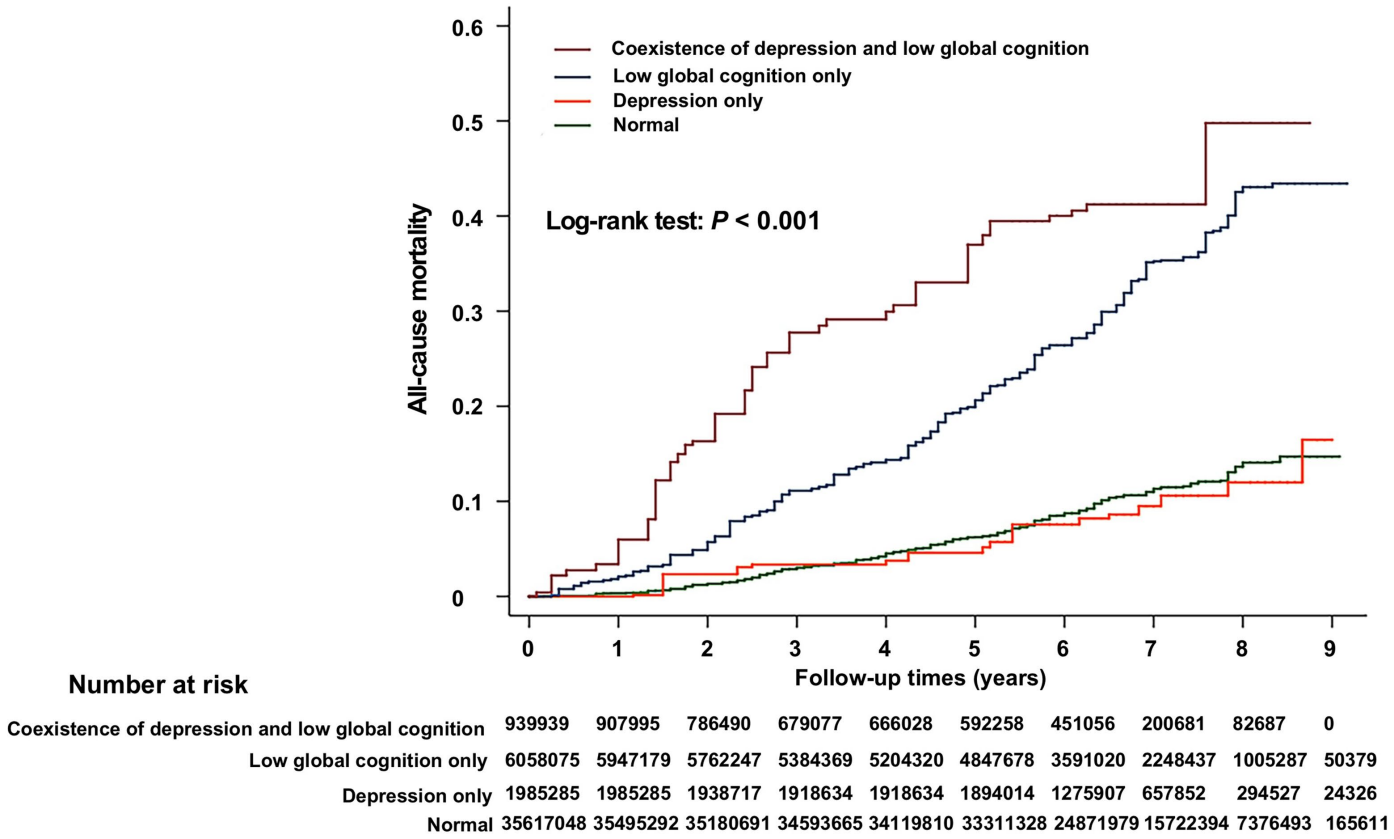
Weighted Kaplan–Meier survival curves of participants with different status of depression and low global cognition. The numbers in the figure are the weighted numbers of at-risk participants.

Weighted Kaplan–Meier survival curves of participants with different status of depression and low global cognition are shown in Figure 1. Among four groups, the coexistence of depression and low global cognition group fared the worst for survival. The joint effects of depression and low global cognition on all-cause mortality, cardiovascular mortality, and cancer mortality are shown in Table 5. Compared with normal groups, low global cognition only and coexistence of depression and low global cognition had higher HRs for all-cause mortality and the HRs were highest in the coexistence of depression and low global cognition group. The HRs for cardiovascular mortality were highest in the coexistence of depression and low global cognition group. No such associations were observed for cancer mortality. Further adjusting for disability in ADLs, IADLs, LSA, LEM, and GPA attenuated these associations, but the HRs for all-cause mortality and cardiovascular mortality remained the highest in the coexistence of depression and low global cognition group.

**Table 5:** Joint effects of depression and low global cognition on all-cause mortality, cardiovascular mortality and cancer mortality.

Mortality	Normal		Depression only		Low global cognition only		Coexistence of depression and low global cognition	
	HR (95% CI)	*P*	HR (95% CI)	*P*	HR (95% CI)	*P*	HR (95% CI)	*P*
All-cause mortality								
Model 1	1.00 (reference)	/	0.89 (0.34, 2.34)	0.808	1.65 (1.18, 2.29)	0.004	3.19 (1.72, 5.93)	0.001
Model 2	1.00 (reference)	/	0.79 (0.30, 2.06)	0.616	1.51 (1.07, 2.13)	0.020	2.31 (1.18, 4.55)	0.017
Cardiovascular mortality								
Model 1	1.00 (reference)	/	0.74 (0.27, 2.00)	0.537	1.69 (0.96, 2.98)	0.069	5.41 (2.01, 14.56)	0.002
Model 2	1.00 (reference)	/	0.65 (0.22, 1.92)	0.421	1.54 (0.83, 2.87)	0.167	4.11 (1.43, 11.75)	0.010
Cancer mortality								
Model 1	1.00 (reference)	/	0.65 (0.18, 2.42)	0.513	1.25 (0.64, 2.47)	0.504	1.14 (0.29, 4.51)	0.852
Model 2	1.00 (reference)	/	0.61 (0.16, 2.34)	0.458	1.21 (0.59, 2.46)	0.595	0.83 (0.17, 4.15)	0.817

Model 1: adjusted for age categories, gender, race, marital status, education, smoke, alcohol drinking, physical activity, ratio of family income to poverty category, body mass index category, coronary heart disease, diabetes, hypertension, stroke and cancer.

Model 2: adjusted for model 1 covariates plus disability in activities of daily living, instrumental activities of daily living, leisure and social activities, lower extremity mobility and general physical activity.

HR, hazard ratio; CI, confidence interval.

## Discussion

The study provides the finding that interactions between depression and low global cognition existed for disability in IADLs and LEM, and cardiovascular mortality. Compared with normal participants, participants with both depression and low global cognition had the highest risk of disability in ADLs, IADLs, LSA, LEM, and GPA. Besides, participants with both depression and low global cognition also had the highest risk of all-cause mortality and cardiovascular mortality, and these associations remained after adjusting for disability in ADLs, IADLs, LSA, LEM, and GPA.

Investigating the combined associations of depression and low global cognition with functional disability and mortality is critical because coexistence of depression and cognitive impairment is common in older adults. The prevalence of coexistence of mild cognitive impairment and depression was 9.1% in older patients with diabetes and 5.1% in older Japanese (Hidaka et al., 2012; Gorska-Ciebiada et al., 2014). Hence, elucidating the joint effects of depression and cognitive impairment on functional disability and mortality is especially helpful for old adults to realize the adverse effect of the coexistence of depression and cognitive impairment, which may prompt them to adopt efficient intervention. Our finding also highlighted the importance of early screening of depression and cognitive impairment in older adults.

The combined associations of depression and low global cognition with functional disability are debated (Mehta et al., 2002; Raji et al., 2002; Li and Conwell, 2009; Olaya et al., 2016; Jarvis et al., 2019; Wu, 2021). Previous studies observed an interaction effect between depressive syndrome and cognitive impairment for disability in IADLs and lower body function (Raji et al., 2002; Li and Conwell, 2009), while other studies found no interaction effect existed (Mehta et al., 2002; Wu, 2021). A study conducted in older adults also found interaction effects between depression and cognitive impairment for disability, but the direction of the interaction was different (Olaya et al., 2016). They found that the risk of disability was higher in participants with depression and good cognitive function than participants with depression and poor cognitive function. Through data analyzed from the national survey, the present study further supported the view that interactions between depression and low global cognition existed for disability in IADLs and LEM. Although no such significant interaction was observed for disability in ADLs, LSA, and GPA, the directions of interaction terms were all positive. Participants with both depression and low global cognition had the highest risk of disability in ADLs, IADLs, LSA, LEM, and GPA. The reason for these non-significant results might be that the sample size of this study is relatively small.

The present study also found that participants with both depression and low global cognition had the highest risk of all-cause mortality and cardiovascular mortality, and interaction between depression and cognitive impairment existed for cardiovascular mortality. Our finding was consistent with some previous studies (Mehta et al., 2003; Georgakis et al., 2016). Considering the combined associations of depression and low global cognition with functional disability and the effect of functional disability on mortality (Murad et al., 2015; Wu et al., 2016), we further explored whether the joint effects of depression and cognitive impairment on mortality were influenced by their effects on functional disability. We found that the HRs of all-cause mortality and cardiovascular mortality remained highest in participants with both depression and low global cognition after adjusting for disability in ADLs, IADLs, LSA, LEM, and GPA, which suggested that the combined effects of depression and cognitive impairment on mortality were independent of functional disability. Our finding suggested that interventions might be needed for older participants with both depression and cognitive impairment in preventing functional disability and premature death.

The reason why interactions between depression and low global cognition existed for IADLs disability, LEM disability, and cardiovascular mortality might be complicated. Depression might lead old adults with cognitive impairment unwilling to engage in physical activity, receive medical treatment, and adopt healthy lifestyles (Raji et al., 2002; Brummett et al., 2009; Li and Conwell, 2009; Jarvis et al., 2019), which may further increase the risk of IADLs disability, LEM disability, and cardiovascular mortality. In addition, older adults with cognitive impairment were more likely to have the poor neurological function, which was closely related to functional disability and cardiovascular mortality. Studies thought that good emotion might be helpful in recovering neurological function and slowing the decline in cognitive performance (Matsunaga et al., 2008; Santos et al., 2013; Stegemoller, 2014; Jarvis et al., 2019). These findings suggested that except for the positive effect itself, good emotion may further reduce the risk of functional disability and cardiovascular mortality by attenuating the adverse impact of cognitive impairment on functional disability and cardiovascular mortality.

We examined the interactions between depression and low global cognition for IADLs disability, LEM disability, and cardiovascular mortality in different age groups, and we only observed significant interactions between depression and low global cognition for disability in LEM in participants aged 80 and above (data not shown). These results should be interpreted in caution because the numbers of participants in different age groups were small in the present study. Further studies with large sample size were needed to determine whether age might influence the interactions between depression and low global cognition for disability in IADLs and LEM, and cardiovascular mortality.

Several strengths existed in the present study. First, this study evaluated several domains of functional disability, and explored the separate and combined associations of depression and low global cognition with each domain of functional disability at the same time. Second, participants of this study were from a national representative survey. Third, a series of covariates were considered in the regression models.

This study has several limitations. First, the causality of depression and cognitive impairment with functional disability was unable to clarify due to the cross-sectional design. Second, the evaluations of depression, cognitive impairment, and functional disability were based on questionnaire survey, which means recall bias might exist in this study. Third, the sample size of this study was relatively small. Fourth, the outbreak of covid19 occurred at the end of the 2019, which might influence the combined associations of depression and cognitive impairment with mortality.

## Conclusion

In conclusion, participants with both depression and low global cognition were more likely to have functional disability, and had the highest risk of all-cause mortality and cardiovascular mortality. Interactions between depression and low global cognition existed for IADLs disability, LEM disability, and cardiovascular mortality. More studies are needed to further explore the associations of depression and low global cognition with functional disability and mortality.

## Data availability statement

The original contributions presented in the study are included in the article/supplementary material, further inquiries can be directed to the corresponding authors.

## Ethics statement

The studies involving human participants were reviewed and approved by the National Center for Health Statistics Research Ethics Review Board. The patients/participants provided their written informed consent to participate in this study.

## Author contributions

SH, YG, and DG designed the study. SH analyzed the data and wrote the manuscript. YG and DG revised the manuscript and supervised the study. All authors read and approved the final version of the manuscript.

## Funding

The present study was supported by grants from the Zhejiang Provincial Natural Science Foundation of China (LQ21C110001), the National Natural Science Foundation of China (82100904), and the Construction Fund of Key Medical Disciplines of Hangzhou (No. OO20200055).

## Conflict of interest

The authors declare that the research was conducted in the absence of any commercial or financial relationships that could be construed as a potential conflict of interest.

## Publisher’s note

All claims expressed in this article are solely those of the authors and do not necessarily represent those of their affiliated organizations, or those of the publisher, the editors and the reviewers. Any product that may be evaluated in this article, or claim that may be made by its manufacturer, is not guaranteed or endorsed by the publisher.
